# P-938. Safety of Short versus Extended Antibiotic Therapy for Neutropenic Fever in Hematopoietic Cell Transplant Patients: A Retrospective Cohort Study

**DOI:** 10.1093/ofid/ofaf695.1141

**Published:** 2026-01-11

**Authors:** Lucy Cai, Leah H Puglisi, Kyle Molina, Miguel Goicoechea

**Affiliations:** Scripps Clinic/Scripps Green, San Diego, CA; Scripps Health, San Diego, California; Scripps Health, San Diego, California; Scripps Health, San Diego, California

## Abstract

**Background:**

Early initiation of broad-spectrum antibiotics reduces morbidity and mortality for hematopoietic cell transplant (HCT) recipients with neutropenic fever, but prolonged antibiotic exposure increases risks of *Clostridioides difficile* infection, graft-vs-host disease, and antibiotic resistance. We compared the safety and effectiveness of short versus extended antibiotic therapy for HCT recipients with neutropenic fever in the immediate post-transplant period.

**Methods:**

We performed a retrospective, single-center cohort study of adult HCT recipients with febrile neutropenia admitted between January 2019 and May 2024 to compare outcomes treated short (≤7 days) versus extended ( >7 days) antibiotic course. Primary composite endpoints were (1) clinical failure (30-day all-cause mortality or ICU admission), and (2) adverse events (*Clostridioides difficile* infection or acute kidney injury [AKI]). Multivariable logistic regression was used to adjust for confounding factors.

**Results:**

Overall, 103 patients (55 short-, 48 extended duration) were included. Most (61.2%) underwent autologous transplantation, most frequently for non-Hodgkin lymphoma (29.1%) and multiple myeloma (25.2%), with 58.3% having moderate comorbidity burden (HCT-CI score 2-4). Mean antibiotic duration was 3.6 vs.11.9 days in short and extended groups, respectively. Adjusted for age, gender, transplant type, infection type, and underlying malignancy, there was no difference in clinical failure between groups (OR [95% CI]: 3.36 [0.49, 27.66]; p = 0.229). While composite adverse events were more common in the extended group, the difference was not significant (OR [95% CI]: 3.78 [0.95, 16.7], p = 0.066). AKI, however, was significantly higher in the extended antibiotic group (OR [95% CI]: 5.33 [2.09,15.06], p = 0.048). Recurrent fever was uncommon, and extended antibiotics were associated with longer hospital stays (OR, 84.66; 95% CI, 2.7 – 40475; p = 0.047).

**Conclusion:**

Shorter antibiotic therapy was associated with similar rates of clinical failure compared to extended courses in HCT patients with neutropenic fever. Shorter antibiotic duration was also associated with lower odds of AKI and reduced hospital stays.

**Disclosures:**

All Authors: No reported disclosures

